# (*E*)-*N*,*N*′-Bis[2-(5-bromo-1*H*-indol-3-yl)eth­yl]-*N*,*N*′-(but-2-ene-1,4-di­yl)bis­(4-methyl­benzene­sulfonamide)

**DOI:** 10.1107/S1600536811041791

**Published:** 2011-10-22

**Authors:** Yongbing Lou

**Affiliations:** a School of Chemistry and Chemical Engineering, Southeast University, Southeast University Road 2, Jiangning District, 211189 Nanjing, People’s Republic of China

## Abstract

In the title compound, C_38_H_38_Br_2_N_4_O_4_S_2_, there is a crystallographic inversion center located at the mid-point of the alkene bond. The dihedral angle between the aromatic ring systems in the asymmetric unit is 87.69 (19)°. In the crystal, adjacent mol­ecules are linked by pairs of N—H⋯O hydrogen bonds, generating *R*_2_^2^(16) loops within [1
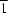
0] chains. Short Br⋯Br contacts [3.6148 (9) Å] are observed between adjacent mol­ecules.

## Related literature

For background to sulfonamides, see: Ozbek *et al.* (2007 ▶). For related structures, see: Abbassi *et al.* (2011 ▶); Akkurt *et al.* (2011 ▶).
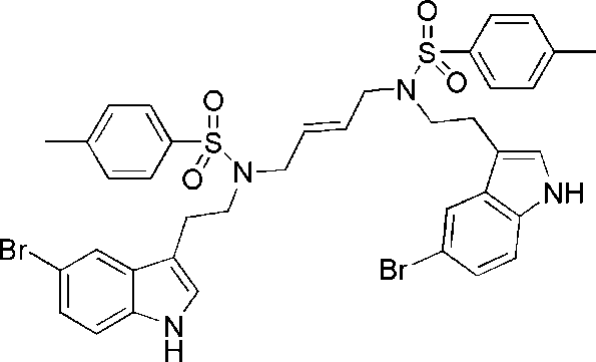


## Experimental

### 

#### Crystal data

C_38_H_38_Br_2_N_4_O_4_S_2_
*M* *_r_* = 838.66
Triclinic, 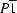

*a* = 5.9222 (8) Å
*b* = 10.4859 (13) Å
*c* = 15.601 (2) Å
α = 79.528 (2)°
β = 87.824 (2)°
γ = 75.186 (2)°
*V* = 921.0 (2) Å^3^
*Z* = 1
Mo *K*α radiation
μ = 2.36 mm^−1^
*T* = 293 K
0.30 × 0.25 × 0.22 mm

#### Data collection

Bruker SMART CCD diffractometer
Absorption correction: multi-scan (*SADABS*; Bruker, 2000 ▶) *T*_min_ = 0.538, *T*_max_ = 0.625
5033 measured reflections
3545 independent reflections
2966 reflections with *I* > 2σ(*I*)
*R*_int_ = 0.015

#### Refinement

*R*[*F* ^2^ > 2σ(*F* ^2^)] = 0.050
*wR*(*F* ^2^) = 0.152
*S* = 1.02
3545 reflections
227 parameters
H-atom parameters constrained
Δρ_max_ = 1.60 e Å^−3^
Δρ_min_ = −1.07 e Å^−3^

### 

Data collection: *SMART* (Bruker, 2000 ▶); cell refinement: *SAINT* (Bruker, 2000 ▶); data reduction: *SAINT*; program(s) used to solve structure: *SHELXS97* (Sheldrick, 2008 ▶); program(s) used to refine structure: *SHELXL97* (Sheldrick, 2008 ▶); molecular graphics: *SHELXTL* (Sheldrick, 2008 ▶); software used to prepare material for publication: *SHELXTL*.

## Supplementary Material

Crystal structure: contains datablock(s) I, global. DOI: 10.1107/S1600536811041791/hb6445sup1.cif

Structure factors: contains datablock(s) I. DOI: 10.1107/S1600536811041791/hb6445Isup2.hkl

Supplementary material file. DOI: 10.1107/S1600536811041791/hb6445Isup3.cml

Additional supplementary materials:  crystallographic information; 3D view; checkCIF report

## Figures and Tables

**Table 1:** Hydrogen-bond geometry (Å, °)

*D*—H⋯*A*	*D*—H	H⋯*A*	*D*⋯*A*	*D*—H⋯*A*
N1—H1*A*⋯O1^i^	0.86	2.05	2.865 (4)	158

Symmetry code: (i) 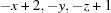.

**Fig. 1.**
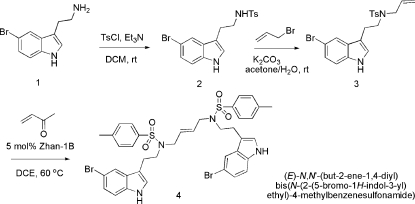
Synthetic route for the title compound.

**Fig. 2.**
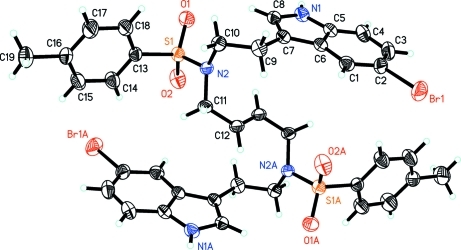
A view of the compound with displacement ellipsoids drawn at the 30% probability level.

**Fig. 3.**
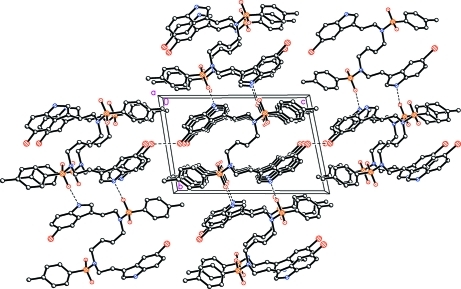
Partial packing view showing the hydrogen bonds network. Hydrogen bonds are shown as dashed lines. For the sake of clarity, the H atoms not involved in the motif have been omitted.

## supplementary crystallographic information

### Comment

Sulfonamides exhibit a broad area of biological activites (e.g. Ozbek *et al.*, 2007). As part of our studies in this area, we now describe the stucture of the title compound, (I) (Fig. 1). For related structures, see: Abbassi *et al.*(2011); Akkurt *et al.*. (2011).

In the title molecule the S atom has a distorted tetrahedral geometry [maximum deviation: O1—S1—O2 =119.82 (19)°] which is possible due to the two S=O double bonds electron repulsion. In the crystal structure, the molecules are linked by four N—H···O hydrogen bonds with adjacent molecules. There also exists weak Br···Br Van der waals interaction to link adjacent molecules.

### Experimental

A solution of 5-bromo tryptamine 1 in (6.68 g, 28.1 mmol) in dichloromethane (100 ml) was cooled in an ice bath, then triethyl amine (8.51 g, 84.3 mmol) and *p*-toluenesulfonyl chloride (5.90 g, 30.9 mmol) were added. The mixture was stirred for 30 min, successively washed with water, brine and dried over MgSO_4_. The solvent was removed in high vacuum, and the tosyl protected tryptamine 2 was obtained in 95% yield (8.38 g, 26.7 mmol) by flash chromatography.

A three-necked flask was charged with tosyl protected tryptamine 2 (8.38 g, 26.7 mmol), acetone (60 ml) and water (60 ml). Then sodium hydroxide (1.60 g, 40.1 mmol) was added. After the solid was dissolved, allyl bromide (3.52 g, 29.4 mmol) was added slowly. The mixture was stirred overnight and evaporated under reduced pressure to remove acetone. The aqueous layer was extracted with CH_2_Cl_2_ (3 × 50 ml). The combined organic phase was washed with brine, separated, dried over Na_2_SO_4_, filtrated, and evaporated under reduced pressure. The residue was purified by recrystallization in ethyl acetate to afford the corresponding allyl indolyl compound 3 as a white solid in 84% yield (7.94 g, 22.4 mol).

A solution of 3 (2.00 g, 5.65 mmol) and methyl vinyl ketone (1.19 g, 16.95 mmol) in 1,2-dichloride ethane (40 ml) was heated to 60 ?, then ruthenium catalyst Zhan-1B (83 mg, 0.113 mmol) was added in one portion. The mixture was stirred for 2 days and evaporated under reduced pressure. The residue was purified by flash chromatography to afford compound 4 in 13% yield (615 mg, 0.73 mmol). Colourless blocks of (I) were grown from ethyl acetate and petroleum solution.

### Refinement

The H atom was placed onto the N atom in indol ring in a calculated positions with N—H = 0.86Å with *U*_iso_(H) = 1.2Ueq(N). The remaining H atoms were placed in a calculated positions with C—H = 0.93–0.97Å and were included in the final cycle of refinement in riding mode with *U*_iso_(H) = 1.2 or 1.5Ueq(C).

### Crystal data

**Table:**

C_38_H_38_Br_2_N_4_O_4_S_2_	*Z* = 1
*M**_r_* = 838.66	*F*(000) = 428
Triclinic, *P*1	*D*_x_ = 1.512 Mg m^−^^3^
Hall symbol: -P 1	Mo *K*α radiation, λ = 0.71073 Å
*a* = 5.9222 (8) Å	Cell parameters from 2384 reflections
*b* = 10.4859 (13) Å	θ = 2.3–26.0°
*c* = 15.601 (2) Å	µ = 2.36 mm^−^^1^
α = 79.528 (2)°	*T* = 293 K
β = 87.824 (2)°	Block, colorless
γ = 75.186 (2)°	0.30 × 0.25 × 0.22 mm
*V* = 921.0 (2) Å^3^	

### Data collection

**Table:**

Bruker SMART CCD diffractometer	3545 independent reflections
Radiation source: fine-focus sealed tube	2966 reflections with *I* > 2σ(*I*)
graphite	*R*_int_ = 0.015
φ and ω scans	θ_max_ = 26.0°, θ_min_ = 2.0°
Absorption correction: multi-scan (*SADABS*; Bruker, 2000)	*h* = −6→7
*T*_min_ = 0.538, *T*_max_ = 0.625	*k* = −10→12
5033 measured reflections	*l* = −16→19

### Refinement

**Table:**

Refinement on *F*^2^	Primary atom site location: structure-invariant direct methods
Least-squares matrix: full	Secondary atom site location: difference Fourier map
*R*[*F*^2^ > 2σ(*F*^2^)] = 0.050	Hydrogen site location: inferred from neighbouring sites
*wR*(*F*^2^) = 0.152	H-atom parameters constrained
*S* = 1.02	*w* = 1/[σ^2^(*F*_o_^2^) + (0.083*P*)^2^ + 0.8618*P*] where *P* = (*F*_o_^2^ + 2*F*_c_^2^)/3
3545 reflections	(Δ/σ)_max_ = 0.001
227 parameters	Δρ_max_ = 1.60 e Å^−^^3^
0 restraints	Δρ_min_ = −1.07 e Å^−^^3^

### Special details

**Table:**

Geometry. All e.s.d.'s (except the e.s.d. in the dihedral angle between two l.s. planes) are estimated using the full covariance matrix. The cell e.s.d.'s are taken into account individually in the estimation of e.s.d.'s in distances, angles and torsion angles; correlations between e.s.d.'s in cell parameters are only used when they are defined by crystal symmetry. An approximate (isotropic) treatment of cell e.s.d.'s is used for estimating e.s.d.'s involving l.s. planes.
Refinement. Refinement of *F*^2^ against ALL reflections. The weighted *R*-factor *wR* and goodness of fit *S* are based on *F*^2^, conventional *R*-factors *R* are based on *F*, with *F* set to zero for negative *F*^2^. The threshold expression of *F*^2^ > σ(*F*^2^) is used only for calculating *R*-factors(gt) *etc*. and is not relevant to the choice of reflections for refinement. *R*-factors based on *F*^2^ are statistically about twice as large as those based on *F*, and *R*- factors based on ALL data will be even larger.

### Fractional atomic coordinates and isotropic or equivalent isotropic displacement parameters (Å^2^)

**Table:**

	*x*	*y*	*z*	*U*_iso_*/*U*_eq_	
Br1	0.15272 (10)	0.49667 (5)	0.09883 (3)	0.0891 (2)	
N1	0.8181 (5)	0.1266 (3)	0.3655 (2)	0.0546 (7)	
H1A	0.9610	0.0811	0.3642	0.065*	
N2	0.4685 (5)	0.2797 (3)	0.60309 (17)	0.0434 (6)	
O1	0.7636 (5)	0.0735 (3)	0.65693 (19)	0.0708 (8)	
O2	0.8105 (5)	0.2919 (3)	0.68103 (19)	0.0727 (8)	
S1	0.66462 (14)	0.20402 (9)	0.67719 (6)	0.0494 (2)	
C1	0.3047 (6)	0.3533 (4)	0.2667 (2)	0.0544 (8)	
H1	0.1549	0.3920	0.2843	0.065*	
C2	0.3727 (8)	0.3809 (4)	0.1820 (3)	0.0619 (10)	
C3	0.5973 (8)	0.3269 (5)	0.1534 (3)	0.0703 (11)	
H3	0.6363	0.3497	0.0953	0.084*	
C4	0.7594 (7)	0.2406 (4)	0.2104 (3)	0.0640 (10)	
H4	0.9097	0.2041	0.1924	0.077*	
C5	0.6929 (6)	0.2093 (3)	0.2959 (2)	0.0493 (8)	
C6	0.4690 (6)	0.2647 (3)	0.3259 (2)	0.0458 (7)	
C7	0.4638 (6)	0.2129 (3)	0.4174 (2)	0.0460 (7)	
C8	0.6779 (6)	0.1281 (3)	0.4379 (2)	0.0486 (7)	
H8	0.7230	0.0783	0.4930	0.058*	
C9	0.2626 (6)	0.2467 (4)	0.4774 (2)	0.0549 (8)	
H9A	0.1424	0.2056	0.4636	0.066*	
H9B	0.1978	0.3430	0.4658	0.066*	
C10	0.3192 (6)	0.2036 (4)	0.5728 (2)	0.0506 (8)	
H10A	0.1750	0.2159	0.6055	0.061*	
H10B	0.3981	0.1089	0.5842	0.061*	
C11	0.3567 (7)	0.4221 (3)	0.6048 (2)	0.0529 (8)	
H11A	0.4049	0.4450	0.6576	0.063*	
H11B	0.1886	0.4349	0.6068	0.063*	
C12	0.4160 (6)	0.5150 (3)	0.5278 (2)	0.0504 (8)	
H12	0.3245	0.6028	0.5184	0.061*	
C13	0.5233 (6)	0.1823 (3)	0.7782 (2)	0.0478 (7)	
C14	0.5337 (8)	0.2638 (5)	0.8365 (3)	0.0664 (10)	
H14	0.6181	0.3284	0.8238	0.080*	
C15	0.4170 (9)	0.2487 (5)	0.9144 (3)	0.0724 (11)	
H15	0.4258	0.3030	0.9545	0.087*	
C16	0.2897 (8)	0.1565 (4)	0.9343 (2)	0.0652 (10)	
C17	0.2818 (11)	0.0765 (5)	0.8747 (3)	0.0921 (17)	
H17	0.1944	0.0134	0.8871	0.110*	
C18	0.3998 (10)	0.0870 (4)	0.7972 (3)	0.0762 (13)	
H18	0.3958	0.0302	0.7582	0.091*	
C19	0.1589 (11)	0.1434 (6)	1.0195 (3)	0.0955 (17)	
H19A	0.1990	0.1994	1.0557	0.143*	
H19B	0.2010	0.0518	1.0489	0.143*	
H19C	−0.0061	0.1708	1.0079	0.143*	

### Atomic displacement parameters (Å^2^)

**Table:**

	*U*^11^	*U*^22^	*U*^33^	*U*^12^	*U*^13^	*U*^23^
Br1	0.1095 (4)	0.0846 (4)	0.0707 (3)	−0.0269 (3)	−0.0398 (3)	0.0043 (2)
N1	0.0450 (15)	0.0586 (17)	0.0567 (17)	−0.0009 (13)	0.0016 (13)	−0.0193 (14)
N2	0.0465 (14)	0.0387 (13)	0.0438 (14)	−0.0101 (11)	0.0100 (11)	−0.0069 (11)
O1	0.0679 (17)	0.0645 (16)	0.0630 (16)	0.0152 (13)	0.0095 (13)	−0.0149 (13)
O2	0.0514 (15)	0.110 (2)	0.0691 (17)	−0.0390 (15)	0.0163 (13)	−0.0249 (16)
S1	0.0411 (4)	0.0573 (5)	0.0472 (5)	−0.0077 (3)	0.0108 (3)	−0.0115 (4)
C1	0.0528 (19)	0.055 (2)	0.057 (2)	−0.0102 (16)	−0.0079 (16)	−0.0168 (16)
C2	0.077 (3)	0.060 (2)	0.050 (2)	−0.0206 (19)	−0.0198 (18)	−0.0068 (17)
C3	0.085 (3)	0.089 (3)	0.044 (2)	−0.033 (2)	0.0050 (19)	−0.0152 (19)
C4	0.062 (2)	0.082 (3)	0.053 (2)	−0.020 (2)	0.0110 (17)	−0.0262 (19)
C5	0.0509 (18)	0.0518 (18)	0.0494 (18)	−0.0128 (15)	0.0015 (14)	−0.0200 (15)
C6	0.0475 (17)	0.0461 (17)	0.0480 (17)	−0.0144 (14)	−0.0007 (13)	−0.0156 (14)
C7	0.0445 (17)	0.0477 (17)	0.0487 (17)	−0.0122 (13)	−0.0004 (13)	−0.0146 (14)
C8	0.0507 (18)	0.0459 (17)	0.0478 (18)	−0.0077 (14)	−0.0003 (14)	−0.0113 (14)
C9	0.0424 (17)	0.069 (2)	0.057 (2)	−0.0168 (16)	0.0026 (15)	−0.0164 (17)
C10	0.0465 (18)	0.0556 (19)	0.0532 (19)	−0.0203 (15)	0.0140 (15)	−0.0111 (15)
C11	0.059 (2)	0.0409 (17)	0.0545 (19)	−0.0064 (15)	0.0200 (16)	−0.0102 (14)
C12	0.0581 (19)	0.0362 (16)	0.0542 (19)	−0.0094 (14)	0.0124 (15)	−0.0070 (14)
C13	0.0492 (18)	0.0492 (18)	0.0422 (17)	−0.0088 (14)	0.0061 (13)	−0.0073 (14)
C14	0.076 (3)	0.078 (3)	0.057 (2)	−0.037 (2)	0.0149 (19)	−0.022 (2)
C15	0.089 (3)	0.082 (3)	0.055 (2)	−0.030 (2)	0.018 (2)	−0.028 (2)
C16	0.081 (3)	0.064 (2)	0.047 (2)	−0.018 (2)	0.0192 (19)	−0.0066 (17)
C17	0.137 (5)	0.091 (3)	0.068 (3)	−0.069 (3)	0.040 (3)	−0.017 (3)
C18	0.119 (4)	0.068 (3)	0.057 (2)	−0.049 (3)	0.024 (2)	−0.018 (2)
C19	0.123 (4)	0.100 (4)	0.064 (3)	−0.035 (3)	0.043 (3)	−0.016 (3)

### Geometric parameters (Å, °)

**Table:**

Br1—C2	1.898 (4)	C9—H9A	0.9700
N1—C5	1.367 (5)	C9—H9B	0.9700
N1—C8	1.377 (4)	C10—H10A	0.9700
N1—H1A	0.8600	C10—H10B	0.9700
N2—C10	1.474 (4)	C11—C12	1.495 (5)
N2—C11	1.476 (4)	C11—H11A	0.9700
N2—S1	1.616 (3)	C11—H11B	0.9700
O1—S1	1.431 (3)	C12—C12^i^	1.309 (7)
O2—S1	1.425 (3)	C12—H12	0.9300
S1—C13	1.762 (3)	C13—C18	1.368 (5)
C1—C2	1.368 (6)	C13—C14	1.369 (5)
C1—C6	1.398 (5)	C14—C15	1.379 (6)
C1—H1	0.9300	C14—H14	0.9300
C2—C3	1.397 (6)	C15—C16	1.358 (6)
C3—C4	1.364 (6)	C15—H15	0.9300
C3—H3	0.9300	C16—C17	1.370 (7)
C4—C5	1.382 (5)	C16—C19	1.515 (5)
C4—H4	0.9300	C17—C18	1.375 (6)
C5—C6	1.404 (5)	C17—H17	0.9300
C6—C7	1.435 (5)	C18—H18	0.9300
C7—C8	1.361 (5)	C19—H19A	0.9600
C7—C9	1.497 (5)	C19—H19B	0.9600
C8—H8	0.9300	C19—H19C	0.9600
C9—C10	1.500 (5)		
			
C5—N1—C8	108.8 (3)	H9A—C9—H9B	107.5
C5—N1—H1A	125.6	N2—C10—C9	112.3 (3)
C8—N1—H1A	125.6	N2—C10—H10A	109.1
C10—N2—C11	115.7 (3)	C9—C10—H10A	109.1
C10—N2—S1	119.1 (2)	N2—C10—H10B	109.1
C11—N2—S1	116.5 (2)	C9—C10—H10B	109.1
O2—S1—O1	119.82 (19)	H10A—C10—H10B	107.9
O2—S1—N2	106.80 (17)	N2—C11—C12	113.2 (3)
O1—S1—N2	106.20 (16)	N2—C11—H11A	108.9
O2—S1—C13	107.97 (17)	C12—C11—H11A	108.9
O1—S1—C13	107.36 (17)	N2—C11—H11B	108.9
N2—S1—C13	108.24 (15)	C12—C11—H11B	108.9
C2—C1—C6	117.5 (3)	H11A—C11—H11B	107.8
C2—C1—H1	121.3	C12^i^—C12—C11	126.4 (4)
C6—C1—H1	121.3	C12^i^—C12—H12	116.8
C1—C2—C3	123.0 (4)	C11—C12—H12	116.8
C1—C2—Br1	118.9 (3)	C18—C13—C14	120.5 (3)
C3—C2—Br1	118.1 (3)	C18—C13—S1	120.1 (3)
C4—C3—C2	120.1 (4)	C14—C13—S1	119.4 (3)
C4—C3—H3	120.0	C13—C14—C15	119.0 (4)
C2—C3—H3	120.0	C13—C14—H14	120.5
C3—C4—C5	117.8 (4)	C15—C14—H14	120.5
C3—C4—H4	121.1	C16—C15—C14	121.8 (4)
C5—C4—H4	121.1	C16—C15—H15	119.1
N1—C5—C4	129.9 (3)	C14—C15—H15	119.1
N1—C5—C6	107.5 (3)	C15—C16—C17	118.0 (4)
C4—C5—C6	122.6 (4)	C15—C16—C19	120.9 (4)
C1—C6—C5	119.0 (3)	C17—C16—C19	121.1 (4)
C1—C6—C7	133.6 (3)	C16—C17—C18	121.7 (4)
C5—C6—C7	107.4 (3)	C16—C17—H17	119.1
C8—C7—C6	106.0 (3)	C18—C17—H17	119.1
C8—C7—C9	127.5 (3)	C13—C18—C17	119.0 (4)
C6—C7—C9	126.5 (3)	C13—C18—H18	120.5
C7—C8—N1	110.3 (3)	C17—C18—H18	120.5
C7—C8—H8	124.9	C16—C19—H19A	109.5
N1—C8—H8	124.9	C16—C19—H19B	109.5
C7—C9—C10	115.5 (3)	H19A—C19—H19B	109.5
C7—C9—H9A	108.4	C16—C19—H19C	109.5
C10—C9—H9A	108.4	H19A—C19—H19C	109.5
C7—C9—H9B	108.4	H19B—C19—H19C	109.5
C10—C9—H9B	108.4		
			
C10—N2—S1—O2	168.7 (2)	C9—C7—C8—N1	−178.1 (3)
C11—N2—S1—O2	−45.2 (3)	C5—N1—C8—C7	−1.3 (4)
C10—N2—S1—O1	39.7 (3)	C8—C7—C9—C10	13.0 (5)
C11—N2—S1—O1	−174.1 (2)	C6—C7—C9—C10	−166.7 (3)
C10—N2—S1—C13	−75.3 (3)	C11—N2—C10—C9	69.3 (3)
C11—N2—S1—C13	70.9 (3)	S1—N2—C10—C9	−144.2 (3)
C6—C1—C2—C3	−1.3 (6)	C7—C9—C10—N2	68.5 (4)
C6—C1—C2—Br1	178.3 (2)	C10—N2—C11—C12	−99.8 (4)
C1—C2—C3—C4	1.1 (6)	S1—N2—C11—C12	112.9 (3)
Br1—C2—C3—C4	−178.4 (3)	N2—C11—C12—C12^i^	−14.2 (7)
C2—C3—C4—C5	0.3 (6)	O2—S1—C13—C18	−170.1 (4)
C8—N1—C5—C4	179.5 (4)	O1—S1—C13—C18	−39.7 (4)
C8—N1—C5—C6	0.5 (4)	N2—S1—C13—C18	74.6 (4)
C3—C4—C5—N1	179.7 (4)	O2—S1—C13—C14	11.4 (4)
C3—C4—C5—C6	−1.4 (6)	O1—S1—C13—C14	141.9 (3)
C2—C1—C6—C5	0.1 (5)	N2—S1—C13—C14	−103.8 (3)
C2—C1—C6—C7	179.9 (4)	C18—C13—C14—C15	−0.2 (7)
N1—C5—C6—C1	−179.7 (3)	S1—C13—C14—C15	178.2 (4)
C4—C5—C6—C1	1.2 (5)	C13—C14—C15—C16	−0.9 (8)
N1—C5—C6—C7	0.5 (4)	C14—C15—C16—C17	0.8 (8)
C4—C5—C6—C7	−178.6 (3)	C14—C15—C16—C19	−178.8 (5)
C1—C6—C7—C8	178.9 (4)	C15—C16—C17—C18	0.5 (9)
C5—C6—C7—C8	−1.3 (4)	C19—C16—C17—C18	−179.9 (6)
C1—C6—C7—C9	−1.3 (6)	C14—C13—C18—C17	1.5 (7)
C5—C6—C7—C9	178.4 (3)	S1—C13—C18—C17	−176.9 (4)
C6—C7—C8—N1	1.6 (4)	C16—C17—C18—C13	−1.7 (9)

Symmetry codes: (i) −*x*+1, −*y*+1, −*z*+1.

### Hydrogen-bond geometry (Å, °)

**Table:**

*D*—H···*A*	*D*—H	H···*A*	*D*···*A*	*D*—H···*A*
N1—H1A···O1^ii^	0.86	2.05	2.865 (4)	158

Symmetry codes: (ii) −*x*+2, −*y*, −*z*+1.
